# A narrative inquiry into the resettlement of armed forces personnel in the Arabian Gulf: a model for successful transition and positive mental well-being

**DOI:** 10.12688/f1000research.75276.2

**Published:** 2022-01-10

**Authors:** Richard Mottershead, Nafi Alonaizi

**Affiliations:** 1 RAK College of Nursing, Ras Al Khaimah Medical and Health Sciences University, Ras Al Khaimah, P.O.Box 11172, United Arab Emirates; 2 Military Medical Services, Military Medical Services, Riyadh, Saudi Arabia

**Keywords:** Keywords: Transition, Mental Well-Being, Resettlement, Retirement, Veteran, Saudi Arabia, Vision 2030

## Abstract

**Background: **The study sought to explore the lived experiences of individuals having served in the Armed Forces of Saudi Arabia, as they made the transition to civilian life and sought new employment opportunities.

**Methods: **Researchers carried out qualitative research in the form of narrative inquiry. Narratives were collected from eleven in-depth interviews conducted in Saudi Arabia in 2021, allowed for insight into participant experiences. Existing literature on military retirement was also investigated.

**Results: **Interviews were transcribed verbatim and analyzed concurrently using thematic analysis to identify patterns or themes. The researchers adopted thematic synthesis as an analytical framework though which descriptive themes from the literature on military retirement were generated. Overall, this approach allowed for the comparison of themes in literature with those of narrative interviews.

**Conclusion: **The study identified challenges encountered by veterans during the resettlement and transitional phase from military to civilian life. There was a general consensus, however, that military life equips individuals with valuable skills that are transferrable to successful post-military employment, known as Positive Transferable Adaptability for Employability (PTAE), ( Mottershead, 2019), which can greatly empower those making the transition. These findings led the researchers to develop a new model for veteran career paths that meet the contemporary employment needs of the Kingdom of Saudi Arabia: the REVERE Transition Model, which identifies six career paths.

## Introduction

Service personnel who retire from the military (hereinafter referred to as “veterans”) have been shown to encounter very different experiences than people who retire from conventional civilian life, and for many veterans, the transition is replete with difficulties and challenges ( Herman & Yarwood, 2015). Military retirement is a challenging life-transition that can severely impact personal identity ( Beech *et al*., 2017) and sense of self, yet the lived experiences of this particular kind of transition are under-researched and not well understood ( Bulmer & Eichler, 2017; Cooper *et al.*, 2017). In the Kingdom of Saudi Arabia (KSA), there is a necessity to explore and establish new knowledge on this very subject. Given the significant financial resources invested in the KSA’s Armed Forces ( Trending Economics, 2021), it is important that policy and practice align to create an effective and empowering plan for resettlement and transition to civilian life and employment. The authors believe that this plan will support and accelerate the KSA’s ability to achieve the nation’s Vision 2030 targets ( Saudi Vision, 2021). The Vision’s aim sets out a blueprint for the country to achieve further economic and social success by 2030. This paper suggests that veterans hold key skills that can help to ensure a vibrant society, a thriving economy, and drive the ambitions of a nation based on effective governing. The purpose of this paper is to explore eleven veterans’ experiences of the transition from military to civilian life as a means of demonstrating that the way in which the KSA’s current system manages veterans’ transition to civilian life is illogical and unsustainable. The authors then suggest a workable solution to this issue that falls outside of current practice in the KSA.

### The draw of military service

Finnegan *et al*. (2010) explain that military service provides a protective “family,” with a community based on shared values, experiences, and socializing. Once an individual has enlisted, the military lifestyle impacts all aspects of their existence. Service also presents them with certain incentives: guaranteed employment, a good and steady income, the potential for a healthy pension, and ample annual leave. Reasonable housing is also available, and when the individual is promoted, accommodation improves. Members are also well-fed, clothed, and are exposed to tremendous adventure, educational training, and physical fitness opportunities. Finnegan *et al*. also highlight the army’s role in providing status and structure, which for those who are seeking a way to hold their lives together, could be a major reason to enlist. The authors go on to explore the fear of losing these benefits in veterans whose alternative is to move into an increasingly fragile economy with uncertain workforce opportunities ( Finnegan *et al*., 2010). This is of particular interest, as issues of employment uncertainty and reduced income are among the themes noted in the present study.

### Identity reconstruction for survival

The reconstruction of one’s identity, as a result of training, socialization, and contact with military culture, allows individual to assimilate into military life. This process is referred to in Goffman’s seminal 1961 work, based on field work in the the United States, as “disculturation”. This involves the internalization of military culture with inculcation of habits, mannerisms, and language, as well as the breakdown of previously held moral boundaries and the assertion of an “adapt and overcome” ethos. Goffman (1961) notes that this ethos is evident in the presence of confidence, courage, and organization in military personnel, and in their suppression of fear.

In addition to the notion of disculturation, Goffman examines the process of joining a “total institution”, a place in which a group of people is actively isolated from the community at large and to which the new identity must adapt (1961). This process certainly occurs in the military context, and as Bradford (2006) global review finds, is more evident during initial basic training, where in its exaggerated form, the process of isolation can produce recruits who are not ruled by their own social and political order, but who are rather self-focused and institutionally blind to the Government or political leader in command. This indoctrination into a military regime, identified as a “self-mortification” process by Goffman, serves to create a new mindset and military identity ( Bradford, 2006). Specifically, participants in Goffman’s study seemed to have established a new identity, which subsequently instigated some radical shifts in their “moral career,” altering their beliefs about themselves, others, and the world around them (1961).

A sociological theory relating to the process of identity reconstruction is provided by Stevenson, who looks specifically at the United States military context. He corroborates the observations made by Goffman and Bradford, from the initial emergence of the individual into basic training, to the remodeling of identity and adoption of a new culture, language, and tradition, which can vary even between different military services ( Stevenson, 2010).

### The dangers of displacement and loss

Parkes and Prigerson (2013) explain that those exposed to military culture can experience such a deep sense of belonging to their military identity and life, that the transition to civilian life is often viewed as a loss. The authors describe the grief associated with the transition as an emotion similar to missing something of significance, and go on to explain that this sense of loss or grief can even occur as a result of losing a valued ability or role ( Parkes & Prigerson, 2013). Parkes (1971) also describes a process, later expanded upon by Rahe (1979), of psychosocial transition in relation to such loss. In his work, Parkes (1971) warns of the dangers of institutionalization and of removing an individual from an institution in which they find comfort. Parkes and Prigerson (2013) state that during such transitions, that which is familiar can suddenly appear to have become unfamiliar. Previously established habits of thought and behavior no longer apply to the new external world, and as a result, confidence is lost within the individual’s internal world.

### Identity and inner conflict

Relevant to the present study is Tajfel and Turner’s Social Identity Theory (SIT), which is used to predict and explain certain behaviors between two or more groups. SIT states that an individual’s self-concept of personal and social identity influences their behavior and actions as they compare themselves to different groups (1986). Ashforth and Mael (1989) argue that this definition allows for an internal cooperative blending of identity, meaning that sense-making and establishing meaning occurs within the individual as they relate to the world around them—subsequently defining and locating their place within the wider context of the social environment. Hockey’s research into British military service personnel provides a concrete example of this: individuals belonging to the group of military personnel are, at their core, trained to be combat-ready through a process of physical and mental training—enabling them to respond to other groups, “threats,” with aggression and violence (1986, 2003).

In keeping with SIT and in relation to the current study, Abrams and Hogg (1990) would argue that the individual veteran *should* be able to locate and define themselves through an evaluation of the social characteristics of the group from which they derive their identity; they stipulate, however, that this process relies on first, defining other groups within their environment and how their identity merges into that perceived reality. According to Erikson’s 1968 seminal work, this can pose a challenge to the veteran whose self-understanding, formed through a perception of norms, attitudes, beliefs, feelings, and behaviors centered within their “ego,” is in conflict with their subjectively defined place within civilian society—a place defined by Brubaker and Cooper (2000) as their “social location”. Continuing along this line of reasoning, Ashforth and Mael’s (1989) research on SIT stipulates that an individual may have multiple roles and belong to numerous groups, thereby creating equally diverse social and personal identities. Of particular relevance here is that while these identities generally function in harmony with each other, when one set of values, norms, behaviors, and beliefs is incompatible with another, a conflict between opposing identities arises within the individual ( Ashforth & Mael, 1989). This can provide significant insight into Saudi veterans’ transition to civilian life.

The literature described here indicates that the military identity is composed of resilient character traits forming a well-developed social identity. There is a notable deficit of references to a policy or practice for dismantling the military identity and allowing for the dramatic shift to civilian attitudes, norms, and feelings. This highlights the timeliness of the present study to examine veterans in the KSA and the need to help them disengage from their military identity and identify with their civilian peers.

### Meaning through belonging

The need to belong is also a feature of SIT. Tajfel and Turner (1986) explain that social identity consists of “those aspects of an individual’s self-image that derive from the social categories to which he perceives himself as belonging”. This claim is bolstered by the findings of Steger and Lopez (2011), who observe a continuing process to establish meaning through belonging. More specific to the current study, Barron, Davies and Wiggins (2008) identify comradeship and associated societal support to be crucial in promoting a sense of belonging for veterans. This is supported by Burnell *et al*. (2006) who cite the importance of comradeship in veterans returning home and transitioning back to post-military identities as civilians. It follows that there is a need to explore post-military identities further within the KSA.

It is evident from the literature on veteran identity, that at its core, is the sense of belonging to something, which extends beyond a uniform and an employment status. The idea of belonging is identified as being central to our understanding of how veterans are given meaning to their lives, and this sense of belonging is intertwined with their successful engagement in a new civilian life. Stillman *et al*. (2009) explain that social relationships are a necessity for individuals to acquire a meaningful life. Additional research by Lambert *et al*. (2010) hypothesized that it is by virtue of relationships that a sense of belonging is created, as they help develop identity and render life more meaningful.

### Financial stress

The literature examined up until this point indicates that there is a need for an international strategy to improve the support available for veterans with mental health needs. Particular concern has been raised by Finnegan *et al*. (2014) over the fact that upon leaving the army, soldiers may face financial and employability challenges for which they may be inadequately skilled to overcome. It is possible, therefore, when considering the research of Finfgeld (2004), that this will inadvertently have negative repercussions on veterans’ mental well-being if there is the absence of an empowering peer-support system. The presence of veteran peer-mentors with a shared military identity could become a powerful therapeutic agent, as it could cross institutional divides and prevent future financial hardships through advice and guidance.

### Coping with change

Seligman’s Locus of Control Theory (2007) corresponds with a key theme that emerged from the participant narratives, as it describes the extent to which a person feels that they have control over the events in their lives, and subsequently their ability to cope. Research undertaken by Solomon and Mikulincer (1990) ascertained that those veterans with a limited or lack of focus or control, were more at risk of developing PTSD. In participating veterans’ accounts, control appeared to have been a key feature of the transitional phase from the Armed Forces to civilian life. There was evidence within their narratives of a link between a lack of control and despondency, that would ultimately result in a negative outcome. This negative outcome ranged from unexpected employment challenges and strife, to loss of identity. Through the thematic analysis of veteran narratives and reflexivity, it was noted that for those veterans who believed they had lost or been deprived of control appeared to develop, what Seligman (1972) identified as, “learned helplessness”. According to the authors, learned helplessness occurs in situations in which the individual has little control over their life, and stress and burnout become major concerns in daily functioning as a result. These observations highlight notion of helplessness as a potential focal point for efforts to help veterans cope with change.

The necessity to cope with change and to understand how veterans can be empowered is a crucial focal point within this this emergent theme. Veteran narratives ascribed meaning and importance to the identity formed through social interactions (shared beliefs, values, and the process of military indoctrination) that took place during their time within the military community. Further, afield, research by Finnegan *et al*. (2014) highlights the disproportionately high rates of depression experienced by veterans in comparison to other professions. It would appear that the military identity creates positive mental well-being but that a detachment can lead to a diminished self-worth and perceived control.

The seminal works of Bowlby (1969, 1980) explains that a state of separation forms the basis for explaining attachment disorder. Bowlby’s research concentrated on the separation of an infant from their mother, creating a state of anxiety for the child which then led to depression and apathy ( 1973). Relevant to this is recent research undertaken by Finnegan *et al.* which highlights how the British Army is predominately composed of young men, often from disadvantaged backgrounds, in which depression is a common mental health disorder. This pre-existing fragile mental state can only be exasperated by a transition to civilian life in what is essentially a state of separation from that which has allowed them to cope ( Finnegan *et al.*, 2014). Within the veteran narratives, there was a suggestion that there were signs and symptoms of depression as the realization of retirement and changing status and financial resources became evident. Finnegan *et al.*’s (2014) work argues that unless the stressors for depression are not better understood, the ramifications will be the incapacitation of supposedly physically fit individuals. The researchers further suggest that this must be related to the poor understanding of the relationship to the causes of depressive symptoms and imbalances with regard to access and treatment ( Finnegan *et al.*, 2014). The participants spoke openly about the difficulties and anxiety they had faced, along with other “problems” (evident in most cases as allusions to mental distress and poor mental health but not always articulated as such), and that they felt that civilians struggled to understand the unique stressors associated with this loss of belonging to the Armed Forces. This self-awareness highlights the mindfulness and self-understanding that the participants had of their reality.

### Self-understanding

Goffman’s (1963) presentation of self-theory can be discussed in terms of veterans, and states that self-understanding, as formed through self-perception of norms, attitudes beliefs, feelings and behaviours centred within ‘self’, is in conflict with their subjectively defined place within civilian society. This can pose a challenge to the military veteran when viewed through the seminal work of Erikson (1968), if their self-understanding, as formed through self-perception of norms, attitudes beliefs, feelings and behaviours centred within ‘ego’, is in conflict with their subjectively defined place within civilian society. A place defined by Brubaker and Cooper (2000) as a ‘social location’.

The corresponding author’s previous research was identified within the 2014 Veteran’s Transitional Review by Lord Ashcroft, the UK’s first transitional review. This government review highlighted that the process of re-entry into a civilian identity can be challenging for some veterans and the evidence appeared to suggest that those veterans lacking in understanding of the transitional process, struggled to adapt to new roles and identities. Likewise, Forces in Mind (2021) has published a policy statement on successful transition to civilian life for service leavers and the message is clear in that an effective transition can be challenging and barriers remain with veterans not always wanting to admit that they may need assistance. Goffman (1963) stipulates through his dramaturgical analysis approach, that an individual may have multiple roles and belong to numerous groups, therefore creating equally diverse social and personal identities. The literature would suggest that the transitional process from military life creates challenges around self-understanding, as civilian values seem to encompass military values in certain circumstances.

The study will seek to draw parallels between these themes of identity reconstruction, the dangers of displacement and the draw of military service, found in existing literature, and the themes that emerge from our study participants’ own narratives. The study presents the rationale for a specific approach that encapsulates the complexity of capturing the lived experience. This approach considers a new source of knowledge, the veterans’ perceptions. These capture both the individual and social experiences of transitioning from military service to civilian life. This study is an attempt to create an understanding through the voices of those that have served KSA, and an effort to empower them within the Saudi Vision 2030.

## Methods

### Ethics

All participants provided written informed consent and that their anonymised data could be used for the purpose of this study. Ethical approval was granted via the authors’ institutions.

This study provides a methodological focus which aims to provide a new understanding of the veterans’ (participants’) lives as they experienced transition from military personnel to civilian. Therefore, the study adopts a qualitative approach. Qualitative research involves the exploration of social interactions between individuals in specific situations and identifying meaning from these interactions ( Denzin & Lincoln, 2005). Qualitative researchers aim to understand the world from the perspective of the research participants, and they do this using various research methods ( Denzin & Lincoln, 2005). Our study used semi-structured interviews which collected narrative data from participants and allowed for observations of their behaviour in specific social settings. There were eleven participants in total who had all served in the KSA Armed Forces (see Table 1).

**Table 1.** Demographic data of the participants.

Code No.	Age	Gender
Veteran 1	49	Male
Veteran 2	51	Male
Veteran 3	48	Male
Veteran 4	51	Male
Veteran 5	55	Male
Veteran 6	48	Male
Veteran 7	49	Male
Veteran 8	50	Male
Veteran 9	52	Male
Veteran 10	52	Male
Veteran 11	50	Male

Narratives provide both a practical and holistic approach to collecting information about another individual’s life, which aids others to glimpse inside their perceived reality and widen our understanding of human existence. In selecting a narrative methodology, the research study sought to allow the veterans to tell their life stories and increase knowledge of their lived experiences of transition from the KSA Armed Forces. According to Bochner (2015), allowing individuals to tell their life stories enables them to make sense of their lives by placing meaning to their experiences in a tangible form. Atkinson (1998) concurs when explaining that narratives allow for the generation of rich subjective meaning that allows individuals to present their life events as they wish them to be presented. Plummer (2001) explains that narrative researcher can enable the participant to create the frame of reference for the telling of the life story and the method highlights the interactions that exist between the individual and their social world. Given the need to create a working strategy to empower KSA military veterans, this methodology was seen to be crucial.

Local COVID-19 restrictions were adhered to for face-to-face interviews or were conducted online to collect the veteran’s narratives from within the Kingdom of Saudi Arabia in 2021. A snowballing sampling method was chosen as a recruitment strategy. Initial participants were selected via NA’s social networks based within Saudi Arabia. This sample technique provided a purposive approach to overcoming the problems associated with sampling this population. Lee (1993) explains that the snowballing technique is particularly useful when attempting to access participants who may be reluctant to share their life stories. The researchers relied upon the assumption that a bond or link exists between the initial sample and others in the same target population, which allowed for a series of referrals to be made to the researchers from within a circle of acquaintances (veterans) as advocated by Berg (1988). Interviews were then conducted at a time and place of the choosing of the participant. Standing (1998) highlights that the researcher have an opportunity to build rapport with participants who have been introduced by other participants, who have already explained the objectives of the study and the way in which it deals with consent and confidentiality.

The process of collecting the narratives occurred between May to September 2021. Interviews were conducted using a semi-structured format, incorporating an interview schedule devised to incorporate themes identified from the thematic synthesis of the literature. Bachman and Schutt (2013) recommend this approach, as it ensures that all dominant themes and main areas of interest are covered, guaranteeing consistency between interviews, whilst also maintaining sufficient flexibility to allow for unanticipated themes to be identified and discussed.

This approach was adopted due to the evidence provided by Bryman (2012), who stated that this strategy encouraged interviewees to fully communicate their experiences through open, free-flowing accounts that could then be explored with prompts and open questions. As suggested by Bryman (2012), this avoids inadvertently leading or influencing participants in their responses and allowed the study to gain a nuanced understanding of the issues under investigation. The questions were the same for online and face-to-face interviews. As recommended by Bryman (2012), a digital recorder was used to record interviews that were face-to-face and the recording facility on a video-conferencing platform, Microsoft Teams, assisted greatly in the interview process. This process allowed both the participant and researchers to relax and converse more freely without the need for extensive notetaking, which can cause distraction and inhibit communication flow.

The narrative interviews were analysed using thematic analysis as utilised by Braun and Clarke (2006). Braun and Clark recommend a thematic analysis be adopted as part of multiple qualitative methods. Thematic analysis is a process of visualising and encoding qualitative research material through the formation of codes and themes. As the sample for this phase of data analysis is relatively small (9), it was decided that manual examination of the data and recurring themes would be appropriate. The use of NVivo ( Hilal & Alabri, 2013) was rejected in line with the recommendations from Silverman (2005), who argues that software programmes for qualitative research are not a substitute for researcher data analysis. Boyatzis (1998) describes a theme as a pattern located within the data that either partially describes and gives order to a feasible observation, or in its entirety, is able to interpret and therefore give meaning of a phenomenon. His process of analysing the narratives was selected due to its flexible and straightforward technique, which created an evidenced theoretical framework that could provide insight into the lived experiences of the veterans.

In utilising a thematic approach in analysis, Braun and Clarke (2006) insist there are several phases of data analysis. This multi-stage approach as advocated by Braun and Clarke (2006) lends itself to a constructionist framework as suggested by Burr (2003). Burr suggests that this approach allows for analysis to include what is unique to a participant, what is shared amongst participants, a description of the experience, an interpretation of the experience, a commitment to understanding the participant’s point of view and lastly, a focus on personal meaning-making within a particular context. The narrative interviews post-recording and transcription were analysed within a Braun and Clarke (2006) 6-stage model.

Phase one included a repetitive reading of all the narrative transcripts. In this research study, the researchers began by reading the interview transcripts several times. This repeated reading allowed the researchers to enter into the veteran’s world and ‘ *listen*’ to them as they gave voice to their beliefs and experiences. At this point, it was decided that the coding for the participants’ interviews would be numerical and a pseudonym would not be used to identify them. The rationale for this decision was based on the belief that the researchers could actively listen to the voices and remember the interview with more clarity.

The researchers believed that by using numbers to code the participants’ identity, there would be a continuity of information from the recordings of the interviews through to the writing up of the transcripts and the notes associated with these interviews. If the coding had been changed to use pseudonyms in the middle of the analysis, it is believed that some of the unspoken meaning of the participants’ experiences would have been lost. Also, clear identification was required in order to allow for in-depth analysis and data triangulation between the participants narratives. Continual use of reflexivity ensured that this interpretation occurred without any undue prejudices, stereotypes or personal attributions from the researcher’s perspective.

Suresh and Chandrashekara (2012) explain that determining the optimal sample size for a study guarantees an adequate ability to detect a degree of significance and is therefore, a critical step in the design of a planned research proposal. Guest, Bunce and Johnson (2006) argue that determining a sample size typically relies on the concept of saturation, meaning the point at which no new information or themes are observed in the data. Malterud, Siersma and Guassora (2016) suggest that there are different sample sizes depending upon the research. Mason (2010) suggest that smaller sample sizes can be useful where a detailed exploration of the phenomenon from several different perspectives can be highly beneficial. Malterud, Siersma and Guassora (2016), however, suggests that there is no exact number and it depends on the individual study and when data saturation has been achieved. Within this study, the researchers ascertained that saturation had been met with the nine participants.

These prejudices, stereotypes or personal attributions were believed to arise from using aliases. When initially the notion of coding was considered, fictitious names were assigned and a list of nine names was constructed. However, each name had a specific meaning or connotation associated with it to the researcher. It was believed that these connotations would be a barrier to the ability of the researcher to be empathic and to immerse themselves into the participant’s world. In effect, while analysing and interpreting the data, the researcher could ‘hear’ the participant’s voice and did not want that voice being ‘confused’ by the voice of someone else that the researcher associated with that pseudonym. At this step, the intention was to focus directly on the veterans and become accustomed to the content of the life stories, the pace and flow of the interview and the relationship between researcher and veteran (participant).

The second phase focused on notetaking of any information within the data that appeared to be relevant to the research questions and amalgamating the thoughts and beliefs from the veterans. This was a more administrative step than the previous step, in that it looked at writing up the life narrative interviews into transcripts and reviewing the written text, in order to affix tenuous links from the data, culminating in beginning the process of clumping the data into codes and subsequent themes ( Braun & Clarke, 2006). For this part of the process, the researcher printed off copies of the transcripts and on each individual transcript, began a line-by-line analysis of the content and then made handwritten reflexive notes in the margin. These notes focused on any pertinent information and used specific key words to identify this information with different coloured highlighting for the codes and emerging themes. At this stage, the notes were mainly descriptive with meaning to the individual explored superficially and not to any depth. Miles and Huberman (1994) suggests that these initial notes should include a description of the content, selecting key words, focussing on language and being aware of conceptualisation of how the participant makes sense of their experiences. This initial process was repeated several times with new documents printed off, updated and re-read, allowing new awareness of the data to be made by the researcher. These exploratory notes were then used to underpin the next stage in the process.

Phase three identified themes that emerged from the interviews and were further analysed in more depth. The exploratory notes made in phase two were used to create new documents in order to develop emergent themes as Braun and Clarke (2006) suggest the focus moves in this phase from the transcript itself to the notes created by the researcher. Again, as for phase two, the documents were printed off several times, each time the notes being refined, and descriptors changed or modified as required in light of how the researcher began to enter into the world of the veteran in more depth. There was, however, a cyclical process that emerged here for the researchers where the focus moved from notes made to an original text as the depth of interpretation increased. At this step the handwritten notes in the margins expanded from specific key words as used in phase one to longer descriptive phrases.

The emphasis in this phase moved slightly away from the veterans-led representation of the data to a more researcher involved enquiry as the data was starting to be analysed. Within this phase, a series of corresponding themes started to emerge from participants narrative interviews and information relating to these themes was colour coded in the transcript documents. By this phase, the data set had become larger with more detailed information to be analysed. Braun and Clarke (2006) suggest that in this stage, the transcripts are de-constructed for analysis and it is possible to establish a thematic map. During this phase, the researcher started to think about the relationship between codes, between themes, and between different levels of themes (e.g., main overarching themes and sub-themes within them).

Phase four signals part of the re-constructing stage and in essence, are explorative in that the aim is to link any connections between themes and a decision made to include or exclude material. In this step, in some instances, phases one to three were repeated. For Patton (2005), the key focus in this phase is discovering patterns and connections between emergent themes. As advised by Patton (2005) the researcher adopted a dual criteria for reviewing the emerging themes through a process of internal homogeneity and external heterogeneity. This is advisable in ascertaining similar themes across the veteran sub-group. Boyatzis (1998) advises that data within themes should cohere together meaningfully, whilst there should be clear and identifiable distinctions between themes. At this point on the analysis phase, the use was also made of a large whiteboard to make the handwritten notes more ‘visible’ to the researchers. The researchers would communicate via videoconferencing and discuss the overall view of the data and emergent themes. This specifically allowed the ‘movement’ of information from one theme to another as it became apparent some quotes aligned more with certain themes than others. If the researchers considered that some of the themes were irrelevant or did not fit in with the research questions, then these were excluded.

At this point in the analysis process, the sub-themes started to develop into main themes. By utilising a whiteboard, a matrix was constructed with the veteran identification code across the top (1 to 9) and the main themes on the left-hand side. The phrases from the transcripts were used in a simplified form to contextualise the sub-themes. This restructuring and reconsidering of the interpretations echo the iterative nature of thematic analysis.

Phase five according to Braun and Clarke (2006), requires the start of the whole process again, however this time looking at the next participant’s transcript (in effect for this research study this process was repeated nine times). However, for this study phase, five to a certain extent was not utilised in the way Braun and Clarke (2006) advocates. Phase five in this study was integrated earlier into the analysis process, as each transcript was part of a cyclical process where some interviews were more detailed and contained more vibrant and emotive content, whilst others were shorter with less involved content. Each individual transcript was subjected to the same four previous phases as outlined above and suitable care was taken to ensure that each individual veteran’s transcript was considered discretely within this process. Each of the veteran’s experiences were considered primarily as an account of how they contextualise these experiences and explain them in their own terms and expressions, in effect, what these experiences of transition mean to them.

The final phase, phase six, according to Braun and Clarke (2006) requires detailed examination of all the emergent themes from all the transcripts and formatting this in a suitable way. Again, this study deviated from the guidelines from this step. For this study, phase six was a re-visitation of all the information gleaned from the narrative transcripts. In this stage, the emphasis was ostensibly focussed on the interpretation of the data. It was at this point, that the sub-themes were investigated in far greater depth. From this in-depth analysis, several issues arose. From exploring the transcripts from a deeper interpretive stance, it was observed that many of the sub-themes inter-linked and were present within both the veteran’s narratives.

In this final phase, this inter-linkage of the main themes between the participants became more prominent. The names of the main themes were changed several times in phase six in order to represent the content of that theme and the shared relevance. At this point in the analysis, it also became apparent that within some of the individual quotes from the veterans’ transcripts, there were sub-themes that could be attributed to more than one main theme. The decision was made to include these into the separate sub-themes even if replicated, however they would be interpreted and analysed within the context of that main theme. The emergent themes were then placed in an order that was logical and coherent to be explored further by the researchers.

Following Braun and Clarke’s (2006) recommended process of thematic analysis of the narratives, numerous words and phrases (codes) emerged that held at their core, a meaning that extended beyond a uniform and an employment status and established an understanding of the reality of life for the study’s participants. The researchers were the only individuals with full access to the recorded narrative interviews and produced transcripts throughout the length of the research study. Each audio recording was password protected, was never duplicated and once the full transcription of the narrative interview was completed, the audio recordings were destroyed. Hardcopies of the transcripts were stored. The researchers would analyse extracts of each other’s interviews to ensure that findings were supported by the data and therefore a true representation to aid accurate and promote rigour. It was necessary to omit quotes and de-identify information pertaining to specific military units in order to guarantee anonymity and confidentiality.

The first author is a veteran and mental health nurse and the second author is currently serving as a military nurse for a country within the Gulf region. There was therefore a need to adopt reflexivity as an essential approach to augment the trustworthiness, integrity, and credibility of the studies professional use of self, to explore the participants’ understanding and interpretations of their experiences. The use of reflexivity seeks to elicit a greater depth in responses from participants aided by the researchers own former and present military identity and thoughtful use of prior theory and researcher expertise, as advocated by Higate and Cameron (2006). Moustakas (1994) advocates that interpretations would be understood to be a collaboration between the researcher and the participant, in order to bring out underlying conditions and hidden objectives of the phenomenon. Atkinson (1998) and Plummer (2001) also argue that communities are dominated by the voice of the dominant group, whilst the knowledge of ‘others’ outside of the group can go unheard or unnoticed.

Finlay (2002) describes reflexivity as “thoughtful conscious self-awareness,” on the part of the researcher. This process was found to be enlightening for the researchers as an awareness became embedded within the researchers of a mindful evaluation of the participants’ responses, intersubjective dynamics between the two prominent identities (military and emerging civilian identity) and subsequent experiences. As nurses, reflection is embedded within effective practice and so often diffuses into parallel aspects of associated life. Meaning, that reflection can become engrained within a nurses life. For the researchers of this study, reflexivity involved a shift in the understanding of how we construct our knowledge from data collection to something objective.

According to Tanggaard (2008), within qualitative research, it is recommended that narrative accuracy checks be undertaken to ensure that a high degree of accuracy, credibility, validity and transferability are established and maintained. The researchers cross-checked with participants the transcripts and their stories to ensure that it was a true representation and interpretation of what they had discussed. Cohen and Crabtree (2008) acknowledge this process within their own research and add that this approach can prevent personal biases from being included within a qualitative study. Higate and Cameron (2006) highlight the risk of the possible presence of pre-conceived notions for military researchers studying a former military population. Therefore, reflective practice was undertaken between the researchers who would review the data to ensure that the data was accurate and unbiased.

## Results and discussion

Military transition is simply the change from military to civilian status. Far from simple, this can be a move from active duty to veteran or to retired life, but for many it is the beginning of a whole new experience. Table 2 outlines the four main themes that emerged from the data analysis.

**Table 2.** Themes that emerged from the data.

Number	Themes
**Theme one**	Attachment to the military uniform
**Theme two**	Losing control and financial stress
**Theme three**	Self-understanding
**Theme four**	Coping with change

### Theme one: Attachment to the uniform

The first theme emerged from narratives in which participants described their experience of the moment their retirement was announced. Participants spoke about their reflection on the concept of retirement and their level of readiness for the forthcoming chapter in their lives. Although they had varied experiences, most veterans felt unprepared for their retirement, which proved to be a challenging step as it was a difficult concept for the majority to grasp. Some experienced feelings of inadequacy and fear in relation to being unsure of how to begin this phase of their life. They had fears and worries about an unknown future and the cessation of an active work life. Military culture had also impacted veterans’ views of themselves and the world, leaving some feeling lonely and deeply sad. Certain individuals described life as being “unbearable” without their uniform, and others stated that coping was an ongoing process that disproved the expression “time heals all wounds.” Being forced to retire led to depression in some veterans, precisely because they did not have the option to refuse it.

Participants spoke about how scared they were after their retirement, as the uniform gave them an extra boost of confidence. Some asked themselves, “Am I going to lose people’s respect without (the uniform)?”. The military environment creates an association in the minds of military personnel between the uniform and empowerment. Such an association is difficult to undo and can leave a lasting impression after retirement. It leads to questions like, “What am I going to do after retirement?” and “How is life going to be without the uniform?”. While most veterans try to keep working after retirement so they can still feel active and productive, they still feel insecure about doing “normal” jobs; as they miss the feeling of empowerment that a uniform affords. This inherent need to belong for the military veterans, is consistent with the themes in previous research literature, of a continual effort to establish meaning through belonging. Indeed, whilst transition into a civilian role involves identity reconstruction, the veteran relies on comradeship and associated societal support to cope with change. This is of importance, as comradeship supports the transitioning back to post-military identities as civilians and to offset dangers associated displacement and loss.

### Theme two: Losing control and financial stress

This theme emerged in instances when participants described their experience at the beginning of their retirement journey and how it had a considerable impact on their family’s financial position. The majority were concerned that the financial allowance they would receive was not sufficient to cover their basic needs. This experience of financial insecurity was for many their first exposure to this loss of control and created uncertainty for their future. They had become reliant on the security offered by their military employment but recognised a need to establish new ways to regain control.

While many veterans look forward to leaving behind the structure and rules of the military, those who have transitioned usually experience a loss of control and purpose, often identifying this loss as the biggest challenge. Participants found it challenging to find a suitable job in civilian life, and had difficulty performing simple tasks. Many of the participants commented that they had never been taught how to “write in a proper, professional manner” while in the military and that they had never participated in administrative work at any point in their military life.

A number of participants felt that they should have had better training in basic skills required for civilian jobs prior to retirement. For example, veteran one viewed military culture as a commitment to do things properly and to do them well, and to always be prepared for what might happen next, except retirement.

“Honestly, I am not prepared just retiring from the military after twenty years. But unless you’ve been there in my shoes, you don’t really totally understand. However, I have been trained to follow commands. I guess discipline and responsibility so I am accepting my retirement.”

In addition to soft skills, veterans also described how they did not learn the importance of proper communication and decision making in situations that required appropriate and professional communication. Some reported that the level of skills they possessed was limited, as there were many activities they were not permitted to practice while serving in the military. Some felt that in their new jobs, they were only entrusted with simple tasks that they already knew how to execute. One participant stated, “some managers […] underestimate us by giving us small tasks […] this is very frustrating.”

This description indicates that certain managers do not provide veterans with an opportunity to develop professionally. It was this feeling of being unappreciated that affected certain participants’ self-confidence. However, some described how they worked within such constraints to create maximum learning opportunities for themselves, adopting a more proactive approach. One veteran stated, “I am still young and (am) actually considering (taking) some courses at the university.” Overall, however, veterans mentioned loss of purpose and identity as the biggest obstacle faced during transition—and the most difficult one to overcome.

### Theme three: Self-understanding

Participants discussed their thoughts on more effective retirement plans, given the feeling that they had not been adequately exposed to skills which would help them to cope with retirement. The most common suggestion was for better cooperation. Other participants explained that, in order to feel confident to undertake their own business, they would require a sense of financial support and security. The military has, to some extent, acknowledged the difficulty associated with the transition from military to civilian life. Some participants demonstrated self-understanding by acknowledging their over-reliance on their military life and experiences and at times, preoccupation with past stories which still had an influence of their sense of self, that did not end with their retirement from military service. Memories are an essential part of everyday personal and professional life. The participants demonstrated their awareness of how their life and its events have had a major role in shaping their individual characteristics. The veterans spoke of how their military experiences have influenced their establishment of self and who they were but also how this had extended to their families.

### Theme four: Coping with change

This study aimed to explore the lived experiences of eleven veterans, to develop a rich conceptual understanding of their transition from military to civilian life. This last theme emerged from perspectives on their overall journey after retirement. The first theme related to the participants’ thoughts at the moment of retirement, and highlighted the fact that most veterans lack the ability to be flexible and overcome the difficulty of leaving their previous life behind to start their new journey. The narratives illustrated the veterans ability to “bounce back” from difficult experiences, and their military experience appeared to have equipped them with an enhanced level of resilience:

“With the absence of professional planning, actually it is not easy unless otherwise veterans have their own network who they can approach on a personnel level. This helps with coping with change but more can be done by veterans department to introduce veterans and highlight their competencies. Resilience is important but … opportunities help us to cope with the change”. Many of us are attached to our military self … we just need to pass this over more easily into this new life, otherwise we feel like we are not in the driving seat …”.

This narrative illustrates the attachment to the military identity and the sense of belonging that the veterans still feel towards that identity. This can create a sense of a lack of control in coping with change. The veterans attribute the establishment of resilience from their military training. Unlike some traits that people are born with, resilience is something that is learned. It involves behaviors, thoughts, and actions that are learned and developed throughout life. Some veterans expressed how it was difficult for them to find a job, as most jobs require a minimum of two years of experience. Others wanted to complete their education.

In this theme, participants provided insights into the barriers encountered during their professional journey. In addition, developing trust among other people plays an important role in shaping a future career, and although veterans’ overall experience was positive in this respect, some reported negative experience centered on a lack of trust. This consequently had an impact on an ability to cope. Participants also spoke about the education program that they had completed and how it did not prepare them sufficiently for the reality of civilian jobs. After leaving the military, the participants considered themselves civilians. However, they also expected to be treated as respected veterans who had served the country—and be accorded the responsibilities that accompany this role. It was also clear from all of the interviews, that a lack of proficiency in English makes communication in today’s world difficult. The challenge of working across a language barrier was the most commonly mentioned challenge among all respondents and obstacle in coping with change.

### The REVERE model: empowering Saudi veterans for Vision 2030

**Figure 1.**
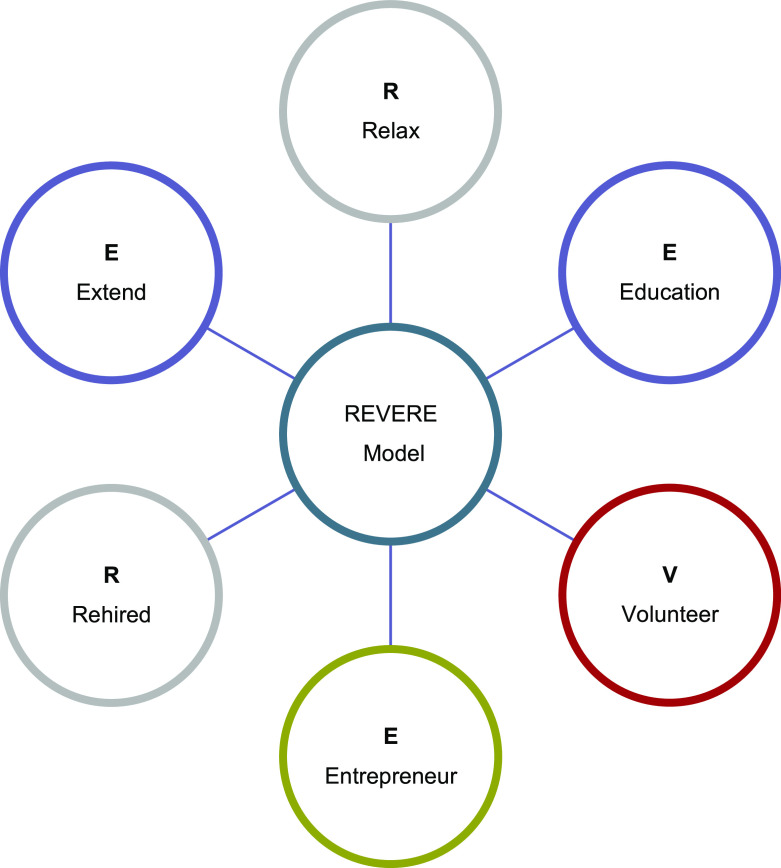
The REVERE model.

The proposed model ( Figure 1) is an adaptation of the Positive Transferable Adaptability for Employability ( Mottershead, 2019) model. The findings of the study, encapsulated within the narratives establishing the four emergent themes, led us to develop a new model to help support a career path that meets contemporary employment needs. The REVERE Transition Model identifies six options for ex-service personnel:

(1) **Relax**: This option maybe the most common choice after retirement.
(2) **Education**: Academic study or professional development.
(3) **Volunteer**: Volunteer with children, teach, or coach sports.
(4) **Entrepreneur**: Self-employment or owning a business.
(5) **Rehired**: Retired military personnel often show leadership skills based on the discipline and training gained in the military, which are ideal to certain civilian careers.
(6) **Extend**: Extension of military service be made by the head of office where strong justification exists, and this happens very rarely.

The REVERE model has been created with a strategic alignment to Saudi Vision 2030. It creates a dovetail between the potential and ability of KSA veterans and the aims of the Vision 2030, to diversify the economy and to meet the growing ambitions of the nation.

### Recommendations for supporting veterans

There is a need for further qualitative narrative research. This study has drawn upon the rich history of narrative methodology to contribute to the research of veterans in the KSA making the transition to a civilian life and identity. Further research into the areas of study listed below are seen by the researchers as necessary to achieving greater insight into the impact of transition and resettlement. This research is also necessary to the potential use of veterans’ skill sets to meet Vision 2030 target. These three suggested areas of study would be incorporated into a national Career Transition Partnership (KSACTP) that would establish a resettlement program, designed to aid veterans as they enter the civilian job market and to make a successful transition to achieve the wider vocational needs of the KSA, that are aligned to 2030.

1) Psychological: Retiring military personnel will need support that recognizes the psychological impact of transition and resettlement on the individual and their family. A positive transition would take into account long-term exposure to a total (military) institution and have mechanisms in place to recondition veterans’ identity and expectations of civilian life and employment.
2) Social: Relatives and or friends should be empowered to participate in the transitional process. The importance of creating a network for retired personnel was evident and seen as a crucially important peer-support initiative. The network aided them to share experiences and emotions, exchanging informational support, and well-being and emotional support that was enhanced via the shared lived experiences.
3) Financial: The study recommends the reduction of the retirement term to 30 years of service, similar to the civilian pathway. Currently the service requirement is 35 years before retirement, and this can prove difficult to achieve. The study recommends a review of the current practice of pension calculation and the implementation of a future retirement plan for new troops, so that they may effectively plan their future earlier on. Career advice and preparation for a successful financial transition could be provided by veterans enlisted to mentor the next generation entering the KSA’s Armed Forces.

### Limitations of the study

The findings of this study do not claim generalizability, having emerged from one geographical region and one homogeneous sample providing their subjective lived experiences, together with the researcher’s own interpretation of them. Undoubtedly, the researcher’s own subjectivity, lived experiences, and research interests may have inevitably influenced the interpretation of these findings as highlighted by Blaikie (2007). However, precautions were taken to limit the impact of this bias where possible, by adhering to a clear and robust methodological framework. Given the deficit of knowledge in this area, while modest, this study represents insight into an under researched topic within the region.

## Conclusion

Four key themes emerged from the research carried out as part of this study, that paralleled themes in existing literature. In narratives of the immediate impact of retirement, participants identified their attachment to the military uniform, which subsequently limited their control and increased their financial stress. This has a direct impact, if only temporary, on their mental well-being. Self-understanding and coping were the most common themes that emerged in narratives of the transition to civilian life. Participants highlighted a major concern regarding a lack resources to help them with this transition but acknowledged that there military skill set was a positive resource that they used to establish resilience and positive mental well-being. In response to this, we have developed the REVERE Model which has the potential to coordinate the strategic integration of veterans into wider society by presenting six options for post-military life. Furthermore, the researchers believe that the mobilization of the veteran community could be a rallying call to meet and the Kingdom of Saudi Arabia’s ambitions of Vision 2030.

## Data availability

Data cannot be shared publicly due to potentially identifiable information. Data will be made available to reviewers or readers via the corresponding author.
