# Supplementary material for: A narrative inquiry into the resettlement of armed forces personnel in the Arabian Gulf: a model for successful transition and positive mental well-being
Source: F1000Res. 2022 Jan 10;10:1290. Originally published 2021 Dec 16. [Version 2] doi: 10.12688/f1000research.75276.2 (PMC8738972; doi:10.12688/f1000research.75276.2)
Supplement: Peer review history

# Peer review: Reviewer response for version 2

**DOI:** 10.5256/f1000research.120112.r119246

**Published:** 2022-01-20

**Authors:** Celia Hynes

**Affiliations:** 1 School of Community Health and Midwifery, University of Central Lancashire, Preston, UK

I found the article engaging and corresponding with other findings on transition. There is a wide range of literature recorded to support the work.

 The methodological approach was appropriate to obtain data in a short space of time and the findings highlight area where changes can be made

 It was interesting to read the areas around identity reconstruction and the link back to Goffman's work. Additionally, the links to attachment theory are also significant as it is recognised that transition is hugely affected by past experiences and future fears.

 Also refreshing to see the REVERE model which will be very applicable. The recommendations are realistic and achievable.

Is the work clearly and accurately presented and does it cite the current literature?

Partly

If applicable, is the statistical analysis and its interpretation appropriate?

Not applicable

Are all the source data underlying the results available to ensure full reproducibility?

Yes

Is the study design appropriate and is the work technically sound?

Yes

Are the conclusions drawn adequately supported by the results?

Yes

Are sufficient details of methods and analysis provided to allow replication by others?

Yes

Reviewer Expertise:

Military veterans / qualitative research

I confirm that I have read this submission and believe that I have an appropriate level of expertise to confirm that it is of an acceptable scientific standard.

# Peer review: Reviewer response for version 1

**DOI:** 10.5256/f1000research.79124.r115475

**Published:** 2022-01-06

**Authors:** Celia Hynes

**Affiliations:** 1 School of Community Health and Midwifery, University of Central Lancashire, Preston, UK

**Editorial Note from F1000Research – 6 ^th^ January 2022:**

**The assigned status of this report has been updated from *Approved with reservations *to *Approved* at the request of the reviewer. No changes have been made to the text of the report.**

 Thank you for inviting me to review this paper which I found very interesting. The paper considers the challenges of transition for military personnel in the Kingdom of Saudi Arabia and this study makes the outcomes comparable to other countries regarding the transition from military service. In particular, the link back to the work of Goffman and also Social identity theory is very good with of course the link to attachment theory.

 I found the abstract clear and I am familiar and comfortable with this style.  

 ​​You will note I have ticked partly for current literature as some of your references are quite dated and there have been more recent papers on transition experience. See for example the Forces in Mind Trust. 

 Also to note that two of your references were not listed - Finnegan *et al., *2017 and Finlay 2002. Further, there are spelling errors for example in the introduction line 9 'Vision' and there are other minor ones so a good proofread is required.

 Overall the design is good with justification given for the sampling choice and the measures taken during the pandemic for interview etc. Data was also collected over a short period of time so again this approach would be necessary.

 The quotes given within the themes - I can only see two - and these are very small font compared to other text.  I would recommend maybe at least one quote per theme. Or remove them and provide overarching, as you have in the others such as 'participants spoke about etc.'

 The REVERE model is good, refreshing, and widely applicable. Recommendations appear achievable. The recognition of how one's self impacts the process appeared to be well considered by the process of reflexivity.

Is the work clearly and accurately presented and does it cite the current literature?

Partly

If applicable, is the statistical analysis and its interpretation appropriate?

Not applicable

Are all the source data underlying the results available to ensure full reproducibility?

Yes

Is the study design appropriate and is the work technically sound?

Yes

Are the conclusions drawn adequately supported by the results?

Yes

Are sufficient details of methods and analysis provided to allow replication by others?

Yes

Reviewer Expertise:

Military veterans / qualitative research

I confirm that I have read this submission and believe that I have an appropriate level of expertise to confirm that it is of an acceptable scientific standard.

# Peer review: Reviewer response for version 1

**DOI:** 10.5256/f1000research.79124.r115477

**Published:** 2022-01-04

**Authors:** Michael Robert Phillips

**Affiliations:** 1 College of the North Atlantic – Qatar, Doha, Qatar

This is an interesting article that has contemporary and future relevance for post-military service and resettlement. Although focused on Saudi Arabia, results and conclusions will inform international resettlement strategies for veterans retiring from a military career. It is well written, argued from fundamental positions, and provides an interesting study of mental health. By assessing four themes results showed challenges and immediate impacts of retirement from the military. The authors used tried and tested techniques, including grounded theory, to make excellent contributions to the literature, e.g. thematic analysis and REVERE Model. In my opinion, the manuscript has many strengths including the provision of a widely applicable method for assessing change. Importantly, it has the potential for incremental progress towards a successful transition and positive mental well-being for resettling armed forces personnel.

 I don't know if it is journal policy, but I would prefer the Abstract to be written as a narrative without being broken down into Sections. Also, it is not usual to include references in the Abstract.

Is the work clearly and accurately presented and does it cite the current literature?

Yes

If applicable, is the statistical analysis and its interpretation appropriate?

Yes

Are all the source data underlying the results available to ensure full reproducibility?

Yes

Is the study design appropriate and is the work technically sound?

Yes

Are the conclusions drawn adequately supported by the results?

Yes

Are sufficient details of methods and analysis provided to allow replication by others?

Yes

Reviewer Expertise:

Human and Physical Geography

I confirm that I have read this submission and believe that I have an appropriate level of expertise to confirm that it is of an acceptable scientific standard.

# Response

**Published:** 2022-01-06

**Authors:** Dr. Richard Mottershead

**Affiliations:** Ras Al Khaimah Medical and Health Sciences University, United Arab Emirates

Dear Professor Phillips,

 On behalf on myself and the co-author we would like to thank you for taking the time to review our research.

 In response to your query over the format of the abstract, this is the proposed style as directed by the journal which we followed diligently.

 Again, thank you for your peer review.

 Dr. Richard Mottershead
